# Identification of donor splice sites using support vector machine: a computational approach based on positional, compositional and dependency features

**DOI:** 10.1186/s13015-016-0078-4

**Published:** 2016-06-01

**Authors:** Prabina Kumar Meher, Tanmaya Kumar Sahu, A. R. Rao, S. D. Wahi

**Affiliations:** Division of Statistical Genetics, ICAR-Indian Agricultural Statistics Research Institute, New Delhi, 110012 India; Centre for Agricultural Bioinformatics, ICAR-Indian Agricultural Statistics Research Institute, New Delhi, 110012 India

**Keywords:** Machine learning, Hsplice, Hybrid approach, Sequence encoding

## Abstract

**Background:**

Identification of splice sites is essential for annotation of genes. Though existing approaches have achieved an acceptable level of accuracy, still there is a need for further improvement. Besides, most of the approaches are species-specific and hence it is required to develop approaches compatible across species.

**Results:**

Each splice site sequence was transformed into a numeric vector of length 49, out of which four were positional, four were dependency and 41 were compositional features. Using the transformed vectors as input, prediction was made through support vector machine. Using balanced training set, the proposed approach achieved area under ROC curve (AUC-ROC) of 96.05, 96.96, 96.95, 96.24 % and area under PR curve (AUC-PR) of 97.64, 97.89, 97.91, 97.90 %, while tested on human, cattle, fish and worm datasets respectively. On the other hand, AUC-ROC of 97.21, 97.45, 97.41, 98.06 % and AUC-PR of 93.24, 93.34, 93.38, 92.29 % were obtained, while imbalanced training datasets were used. The proposed approach was found comparable with state-of-art splice site prediction approaches, while compared using the bench mark NN269 dataset and other datasets.

**Conclusions:**

The proposed approach achieved consistent accuracy across different species as well as found comparable with the existing approaches. Thus, we believe that the proposed approach can be used as a complementary method to the existing methods for the prediction of splice sites. A web server named as ‘HSplice’ has also been developed based on the proposed approach for easy prediction of 5′ splice sites by the users and is freely available at http://cabgrid.res.in:8080/HSplice.

## Background

Exon–intron and intron–exon boundaries in genes are called splice sites, where the former one is donor splice site and the latter one is acceptor splice site [1]. In majority, the donor and acceptor splice sites are conserved with dimer GT and AG at the beginning and at the end of introns respectively [2]. Prediction of splice sites is vital for genome annotation because the accuracy of gene finding programs depend upon the correct identification of true splice sites [1, 3, 4]. However, the conserved GT/AG is not sufficient to locate the true splicing signal, due to the presence of large number of GT/AG di-nucleotides (false positive cases) in genes [1, 5].

Several computational methods have been proposed for the prediction of splice sites, and those can be broadly categorized into two classes, namely, probabilistic approach and machine learning based approach [6]. As far as prediction accuracy is concerned, machine learning approaches are more successful as compared to the probabilistic approaches [1]. In machine learning based approaches, splice site sequences are first transformed into numeric vectors before being used as input in the classifiers [5]. Further, in the class of machine learning approaches, support vector machine (SVM) has been used more successfully for the prediction of splice sites [4]. Baten et al. [7] generated the features based on first order Markov model and used them as input in SVM for splice site prediction by applying polynomial kernel. The difference in the di-nucleotide frequencies between true and false splice sites are also used as features for the prediction of splice sites using SVM with RBF kernel [8]. In another study, the weighted degree (WD) [9] and weighted degree shift (WDS) [10] kernels are successfully used for splice site prediction through support vector machines [3]. Recently, Golam Bari et al. [1] employed SVM with polynomial and RBF kernels for splice site prediction using nucleotide density features. Besides SVM, the Naïve Baye’s classifier has also been successfully used by Kamath et al. [2] for the prediction of splice sites in which an automated feature generation program has been developed.

Feature generation and selection of informative features play a pivotal role as far as the classification performance of machine learning approach is concerned [4]. In most of the feature generation procedures, like MM1 encoding [7], FDTF encoding [8], Baye’s feature mapping [11], nucleotide density based encoding [1], the number of features increases with increase in the length of sequence. On the other hand, features generated by using suboptimal sequence length may provide less discriminatory information for classification of true and false splice sites using kernel based method [3]. Thus, development of an approach that could provide consistent accuracy across different species by using short sequence motifs became the motivation.

Keeping above in mind, an attempt has been made to develop a new computational approach for donor splice site prediction. Initially, positional, compositional and dependency features were extracted for the true and false splice sites. Positional and dependency features were similar to the scores of earlier probabilistic approaches i.e., WMM [12], WAM [13], Shapiro-Senapathy [14] scores and SAE scores [6]. The compositional features were nothing but the composition of di-nucleotides, triplets and tetramers. Out of all generated features, only informative features selected through F-score [15] were retained and used them as input in SVM for classification. By using sequence motif of 15 nt long, the proposed approach achieved consistent accuracy in four species viz, human, cattle, fish and worm. Also, the developed approach was found to be comparable with the existing splice site prediction methods, while compared using an independent test dataset.

## Methods

### Collection and processing of splice site data

Splice site datasets of *Homo sapiens* (HS), *Bos taurus* (BT), *Danio rario* (DR) and *Caenorhabditis elegans* (CE) were considered to evaluate the performance of the proposed approach. Besides, the bench mark NN269 splice site dataset was used to compare the performance of the proposed approach with the other splice site prediction approaches.

Both true and false HS splice sites were collected from HS3D [16] available at http://www.sci.unisannio.it/docenti/rampone/. The true and false sets contain 2796 and 90,923 sequences respectively. Each sequence in the dataset is of 140 nt long having conserved di-nucleotide G and T at 71st and 72nd positions respectively. This dataset has also been used in recent study by Wei et al. [5].

The true and false CE splice sites were collected from http://www.cs.gmu.edu/~ashehu/sites/default/files/tools/EFFECT_2013/UdayProjectDataFiles/SPLICE-SITE/WORM/DonorData.dat. In case of CE, true set contains 1000 sequences and false set contains 19,000 sequences, where each sequence is of 141 nt long having conserved di-nucleotide G and T at 63rd and 64th positions respectively. This dataset has also been used earlier by Kamath et al. [2] for the prediction of splice sites.

In case of BT and DR, exon and intron sequences were collected from UCSC genome browser (https://genome.ucsc.edu/). Using the co-ordinates of exons, true splice sites of length 38 nt (8 nt on exon-end and 30 nt at intron-start) were extracted keeping conserved di-nucleotide G and T at 9th and 10th positions respectively. Further, the false splice site sequences of length 38 nt were randomly extracted from exonic and intronic regions keeping G and T at 9th and 10th positions respectively. In both BT and DR, 10,000 sequences of true and 10,000 sequences of false sites were extracted.

NN269 dataset [17] is a bench mark splice site dataset, which has been extracted from 269 human genes. It consists of 1324 confirmed donor splice site sequences and 4922 false splice site sequences, where each sequence is of 15 nt length having GT at 9th and 10th positions respectively. This dataset has been partitioned into training and test sets consisting of 5256 (1116 true + 4140 false) and 990 (208 true + 782 false) sequences respectively. The dataset is available at http://www.cs.gmu.edu/~ashehu/sites/default/files/tools/EFFECT_2013/data.html. This dataset has also been used in several studies [2, 3, 6] for comparative analysis of splice site prediction methods.

Similar to NN269 dataset, the length of splice site sequences in other species were also restricted to 15 nt. One of the advantages of using shorter length sequence is that the short reads generated from NGS technologies can also be used for determining the splicing junction that helps in improving the alignment quality of short reads [18].

### Feature extraction

Splice site sequences are in the form of strings but machine learning classifier takes numerical features as input. Thus it is required to transform the sequences into numerical feature vectors before using them as input in machine learning classifiers [11]. In this study five different categories of features were used. The five different categories of features are explained as follows:

1. Positional features (P) extracted by using only true splice sites (T): P×T.
2. Positional features (P) extracted by using both true and false splice sites (TF): P×TF.
3. Dependency features (D) extracted by using only true splice sites (T): D×T.
4. Dependency features (D) extracted by using both true and false splice sites (TF): D×TF.
5. Compositional features (C) extracted for each sequence independently (I): C×I.

#### Positional features

Let $p^{t} (\alpha_{i})$ and $p^{f} (\alpha_{i} )$ be the frequencies of nucleotide $\alpha$ at *i*th position in true and false splice site datasets respectively, where $\alpha \in \{ A,C,G\,,\,T\} \,\,$ and $i = 1,2, \ldots ,L$(length of the sequence). The frequencies of nucleotides can be obtained from the frequency matrix of nucleotides [15]. Then, the positional features (*f*) for any sequence are defined as follows:

$$f_{1}^{P \times T} = \sum\limits_{i = 1}^{L} {\log_{2} p^{t} (\alpha_{i} )} \,;\quad\alpha \in \{ A,C,G,T\} ,\quad f_{2}^{P \times TF} = \sum\limits_{i = 1}^{L} {\log_{2} p^{t} (\alpha_{i} )} \,\, - \sum\limits_{i = 1}^{L} {\log_{2} p^{f} (\alpha_{i} )} \,;\quad\alpha \in \{ A,C,G,T\}$$

$$f_{3}^{P \times T} = 100 \times \frac{{\sum\nolimits_{i = 1}^{L} {p^{t} (\alpha_{i} )\, - M^{t} } }}{{M^{t} - N^{t} }}\,;\quad\alpha \in \{ A,C,G,T\} ,$$

$$f_{4}^{P \times TF} = 100 \times \left[ {\frac{{\sum\nolimits_{i = 1}^{L} {p^{t} (\alpha_{i} )\, - M^{t} } }}{{M^{t} - N^{t} }}\,\, - \frac{{\sum\nolimits_{i = 1}^{L} {p^{f} (\alpha_{i} )\, - M^{f} } }}{{M^{f} - N^{f} }}} \right]\,;\quad\alpha \in \{ A,C,G,T\} ,$$

where *M* is the sum of highest frequencies of nucleotides at position 1 to *L* and *N* is the sum of lowest frequencies of nucleotides at position 1 to *L* of splice site motif. The feature $f_{1}^{P \times T}$ is similar to the WMM score [12], $f_{2}^{P \times TF}$ is the difference between such scores obtained by using true and false splice sites, $f_{3}^{P \times T}$ is Shapiro-Senapathy score [14] obtained using true splice sites only and $f_{4}^{P \times TF}$ is the difference in Shapiro-Senapathy score obtained from true and false splice sites.

#### Dependency features

Let $p^{t} (\alpha_{i} \left| {\beta_{j} } \right.)$ and $p^{f} (\alpha_{i} \left| {\beta_{j} } \right.)$ be the frequencies of the nucleotide $\alpha$ at *i*th position given that the nucleotide $\beta$ occurs at *j*th position for the true and false splice site datasets respectively. Then, the dependency features for any sequence are defined as follows:

$$f_{5}^{D \times T} = \sum\limits_{i = 1}^{L} {\sum\limits_{j = 1( \ne i)}^{L} {\log_{2} p^{t} (\alpha_{i} \left| {\beta_{j} } \right.)} } \,;\quad\alpha ,\,\beta \in \{ A,C,G,T\} ,$$

$$f_{6}^{D \times TF} = \sum\limits_{i = 1}^{L} {\sum\limits_{j = 1( \ne i)}^{L} {\log_{2} p^{t} (\alpha_{i} \left| {\beta_{j} } \right.)} } \,\, - \,\sum\limits_{i = 1}^{L} {\sum\limits_{j = 1( \ne i)}^{L} {\log_{2} p^{f} (\alpha_{i} \left| {\beta_{j} } \right.)} } \,;\quad\alpha ,\,\beta \in \{ A,C,G,T\}$$

$$f_{7}^{D \times T} = 2L(L - 1) - 2\sum\limits_{i = 1}^{L} {\sum\limits_{j = 1( \ne i)}^{L} {p^{t} (\alpha_{i} \left| {\beta_{j} } \right.)} } ,\quad f_{8}^{D \times TF} = 2\sum\limits_{i = 1}^{L} {\sum\limits_{j = 1( \ne i)}^{L} {p^{f} (\alpha_{i} \left| {\beta_{j} } \right.)} } - 2\sum\limits_{i = 1}^{L} {\sum\limits_{j = 1( \ne i)}^{L} {p^{t} (\alpha_{i} \left| {\beta_{j} } \right.)} }$$

The feature $f_{5}^{D \times T}$ is similar to the WAM score [12], $f_{6}^{D \times TF}$ is the difference between such scores obtained by using true and false splice sites, $f_{7}^{D \times T}$ is SAE score [6] obtained from true splice sites only and $f_{8}^{D \times TF}$ is the difference in SAE score obtained by using true and false splice sites.

#### Compositional features

Three different types of compositional features i.e., composition of di-nucleotides, triplets and tetramers were used. For a given splice site sequence of length *L*, let $n(\alpha_{1} \alpha_{2} \alpha_{3} \ldots )$ be the number of times the string $\alpha_{1} \alpha_{2} \alpha_{3} \ldots$ occurs in the sequence, by shifting one nucleotide position at a time. The three different compositional features are then computed as follows:

$$f_{9}^{C \times I} (\alpha_{1} \alpha_{2} ) = \frac{{n(\alpha_{1} \alpha_{2} )}}{L - 1};\,\alpha_{1} ,\alpha_{2} \in \{ A,C,G,T\} ;\quad f_{10}^{C \times I} (\alpha_{1} \alpha_{2} \alpha_{3} ) = \frac{{n(\alpha_{1} \alpha_{2} \alpha_{3} )}}{L - 2};\,\alpha_{1} ,\alpha_{2} ,\alpha_{3} \in \{ A,C,G,T\}$$

$$f_{11}^{C \times I} (\alpha_{1} \alpha_{2} \alpha_{3} \alpha_{4} ) = \frac{{n(\alpha_{1} \alpha_{2} \alpha_{3} \alpha_{4} )}}{L - 3};\,\alpha_{1} ,\alpha_{2} ,\alpha_{3} ,\alpha_{4} \in \{ A,C,G,T\}$$

There are 16 di-nucleotides, 64 triplets and 256 tetramers compositions possible. Thus, in total 344 features (4 Positional + 4 Dependency + 336 Compositional) were generated for each splice site sequence.

### Feature selection

From a set of large number of features, selecting a subset of non-redundant features that can potentially discriminate the true and false classes is a preprocessing step in every classification techniques [19]. It helps to reduce (1) the dimensionality of features, (2) memory allocation, and (3) computational time [20]. In this study, F-score [21] was used for selecting important features out of 344 number of features. Feature selection was done in HS3D dataset and same set of selected features were retained in other species also. The F-score for any feature was computed as follows:

Let $\bar{x}_{j}^{ + }$($\bar{s}_{j}^{ + }$) and $\bar{x}_{j}^{ - }$($\bar{s}_{j}^{ - }$) be the mean (standard deviation) values of the *j*th feature for the true and false splice sites respectively. Then, the F-score for the *j*th feature was computed as

$$F(j) = \left| {\frac{{\bar{x}_{j}^{ + } - \bar{x}_{j}^{ - } }}{{\bar{s}_{j}^{ + } - \bar{s}_{j}^{ - } }}} \right|.$$

This approach has also been used in earlier study [15] for feature selection in the area of splice site prediction.

### SVM classification

SVM [22] was employed for prediction purpose because it is non-parametric and most widely used supervised learning technique in bioinformatics, attributed to its sound statistical background [23]. It has been successfully applied for the prediction of several functional elements like translation initiation sites [24], transcription factor-binding sites [25] etc. The predictive ability of SVM is largely dependent upon the type of kernel function that maps the input data to a high-dimensional feature space, where the observations belong to different classes are linearly separable by the optimal separating hyperplane (OSH). To implement the SVM classification, the *svm* function of *e1071* package of R-software [26] was used.

### Cross validation

Cross-validation procedure has been widely accepted for assessing the performance of classifiers on test data set [27]. Thus, a fivefold cross-validation was applied for evaluating the performance of the classifier. To do this, true and false splice site datasets were randomly partitioned into five subsets, and then five sets were created with each set containing a randomly selected subset from both the classes. In each fold of the cross validation, four out of five sets were used for training and the remaining one set was used for testing. This process was repeated five times in such a way that each set was used once for testing.

### Performance measure

Area under receiving operating characteristic curve (AUC-ROC) has been widely used to evaluate the performance of the classifiers [7]. Thus, it was used to measure the prediction accuracy of the proposed approach. The false positive rate (*α*) and true positive rate (1 − *β*) were computed across a range of threshold values lying between 0 and 1. Then the values of AUC-ROC was estimated using the formula $\sum\limits_{i} {\left\{ {\left( {1 - \beta_{i} .\Delta \alpha } \right) + \frac{1}{2}\left[ {\Delta (1 - \beta ).\Delta \alpha } \right]} \right\}}$ [28], where $\Delta (1 - \beta )$ = $(1 - \beta_{i} ) - (1 - \beta_{i - 1} )$, $\Delta \alpha = \alpha_{i} - \alpha_{i - 1}$ and *i* = 1,2,…, *N* (number of test instances). For the imbalanced class distribution, area under precision-recall curve (AUC-PR) provides a better measure for assessing the performance of the classifiers as compared to AUC-ROC [3]. Therefore, the value of AUC-PR was also computed to evaluate the performance of the SVM classifier. The AUC-PR was computed as per Davis-Goadrich approach [29]. A subroutine in R programming language was written to compute AUC-ROC and AUC-PR.

### Kernel selection and parameter setting

Initially, best fitted kernel was chosen out of four different kernels i.e., linear, polynomial, sigmoid and radial basis function (RBF), with default parameter setting. Then, the parameter of the best fitted kernel was optimized. The best fitted kernel was chosen on the basis of ROC curves. The optimum value of parameter for the best fitted kernel was chosen on the basis of highest value of AUC-ROC. For selecting the kernel and optimizing the parameter of the selected kernel, a sample dataset consists of 1000 true and 1000 false sites (randomly selected from HS3D dataset) was used.

### Balanced training and testing datasets

For balanced case, the number of true and false splice sites was kept in the ratio of 1:1 and the datasets for different species were prepared as follows:

*Human*: Ten sets were created with each set containing all the 2796 true splice sites and a subset of 2796 false splice sites. The subsets of false splice sites were randomly drawn from the available false splice sites.

*Cattle*: Ten sets were created with each set containing 5000 true and 5000 false splice sites, randomly selected from the available true and false splice sites.

*Fish*: The datasets were prepared similar to *Cattle* as explained above.

*Worm*: Ten sets were created with each set containing all the 1000 true splice sites and a subset of 1000 false splice sites. The subsets of false splice sites were randomly selected from the available false sites.

### Imbalanced training and testing datasets

For imbalanced case, the number of true and false splice sites was kept in the ratio of 1:5 for human, cattle and fish, which is similar to the proportion of true and false sites present in NN269 dataset. For worm dataset, the ratio was kept at 1:19 as followed in earlier studies [2, 3]. The datasets were prepared in the following ways:

*Human*: Ten sets were created with each set containing all the 2796 true splice sites and a subset of 13,980 (5 × 2796) false sites. The subsets of the false splice sites were randomly selected from the available false splice sites.

*Cattle*: Ten sets were created with each set containing a subset of 1000 true and 5000 false sites, where the subsets were randomly selected from the available true and false sites respectively.

*Fish*: The datasets were prepared similar to *Cattle*.

*Worm*: Ten sets were created with each set containing a subset of 500 true sites and 9500 false splice sites randomly selected from the available true and false sites respectively.

In both balanced and imbalanced situation, the performance of the SVM classifier was measured in terms of AUC-ROC and AUC-PR averaged over 50 sets (10 sets with fivefold in each set), in each species.

### Comparison with other prediction methods

The proposed approach was compared with the state-of-art splice site prediction methods by using an independent test dataset i.e., NN269 [17]. This dataset has been used in several earlier studies on splice site prediction [1–4, 6]. The performance of the proposed approach was compared with that of SVM with MM1 encoding (MM1-SVM) [7], SVM with weighted degree kernel(WD-SVM) [3], SVM with locally improved kernel (LIK-SVM) [3], SVM with weighted degree shift kernel (WDS-SVM) [3] and EFFECT [2]. In MM1-SVM, features are generated based on first order dependency and then used as input in SVM for classification of true and false splice sites. In locally improved kernel, correlations among local subsequences within a small window around a fixed nucleotide position are taken into account. The scores of each such window are summed up to give a weight to that sequence. This weighting scheme emphasizes on important regions of the sequence. The weighted kernel emphasizes on the position dependent information and the weighting decreases the influence for higher order matches between the sequences. In case of weighted degree shift kernel, weights are assigned on shifting of the sequence in either direction. EFFECT uses a two-stage process, where a set of candidate sequence-based features are constructed in the first stage and then the most effective subset is selected for the classification. Both stages make heavy use of evolutionary algorithms to efficiently guide the search towards informative features capable of discriminating true and false splice site sequences. The comparison among the methods was made in terms of AUC-ROC and AUC-PR.

## Results

### Feature selection analysis

Out of 344 features (described under feature selection section), 49 features were obtained with F-value ≥1.25 and rest of the features were having almost similar F-values (i.e., ≪1.25). Therefore, these 49 features were only considered for further analysis. The list of selected features is provided in Table 1. Out of 49 selected features, four were positional, four were dependency and 41 were compositional features. Further, among 41 compositional features, 14 were composition of di-nucleotides, 15 were composition of tri-nucleotides (triplets) and 12 were composition of tetramers. The positional and dependency features were found to have higher F-values as compared to the compositional features. Further, the positional and dependency features computed from both true and false sites were found to have higher F-values than that of computed from true sites only.

**Table 1:** List of selected features using F-score

Feature type	**#**Features	Features
Positional	4	$f_{1}^{P \times T} ,f_{2}^{P \times TF} ,f_{3}^{P \times T} ,f_{4}^{P \times TF}$
Dependency	4	$f_{5}^{D \times T} ,f_{6}^{D \times TF} ,f_{7}^{D \times T} ,f_{8}^{D \times TF}$
Compositional	41	$$\begin{aligned} f_{10}^{C \times I} (AA),f_{10}^{C \times I} (AC),f_{10}^{C \times I} (AG),f_{10}^{C \times I} (CA),f_{10}^{C \times I} (CC),f_{10}^{C \times I} (CT),f_{10}^{C \times I} (GA) \hfill \\ f_{10}^{C \times I} (GC),f_{10}^{C \times I} (GG),f_{10}^{C \times I} (GT),f_{10}^{C \times I} (TA),f_{10}^{C \times I} (TC),f_{10}^{C \times I} (TG),f_{10}^{C \times I} (TT) \hfill \\ \end{aligned}$$ $$\begin{aligned} f_{11}^{C \times I} (AAG),f_{11}^{C \times I} (AGG),f_{11}^{C \times I} (AGT),f_{11}^{C \times I} (CAG),f_{11}^{C \times I} (GAG), \hfill \\ f_{11}^{C \times I} (GGG),f_{11}^{C \times I} (GGT),f_{11}^{C \times I} (GTA),f_{11}^{C \times I} (GTC),f_{11}^{C \times I} (GTG), \hfill \\ f_{11}^{C \times I} (TAA),f_{11}^{C \times I} (TGA),f_{11}^{C \times I} (TGC),f_{11}^{C \times I} (TGG),f_{11}^{C \times I} (TGT) \hfill \\ \end{aligned}$$ $$\begin{aligned} f_{12}^{C \times I} (AAGG),f_{12}^{C \times I} (AGGT),f_{12}^{C \times I} (CAGG),f_{12}^{C \times I} (GAGG), \hfill \\ f_{12}^{C \times I} (GGGT),f_{12}^{C \times I} (GGTA),f_{12}^{C \times I} (GGTG),f_{12}^{C \times I} (GTAA), \hfill \\ f_{12}^{C \times I} (GTGA),f_{12}^{C \times I} (GTGG),f_{12}^{C \times I} (TAAG),f_{12}^{C \times I} (TGAG), \hfill \\ \end{aligned}$$

Out of 344 generated features, 49 features are selected among which four are positional, four are dependency and 41 are compositional features

### Kernel and parameter analysis

**Fig. 1:**
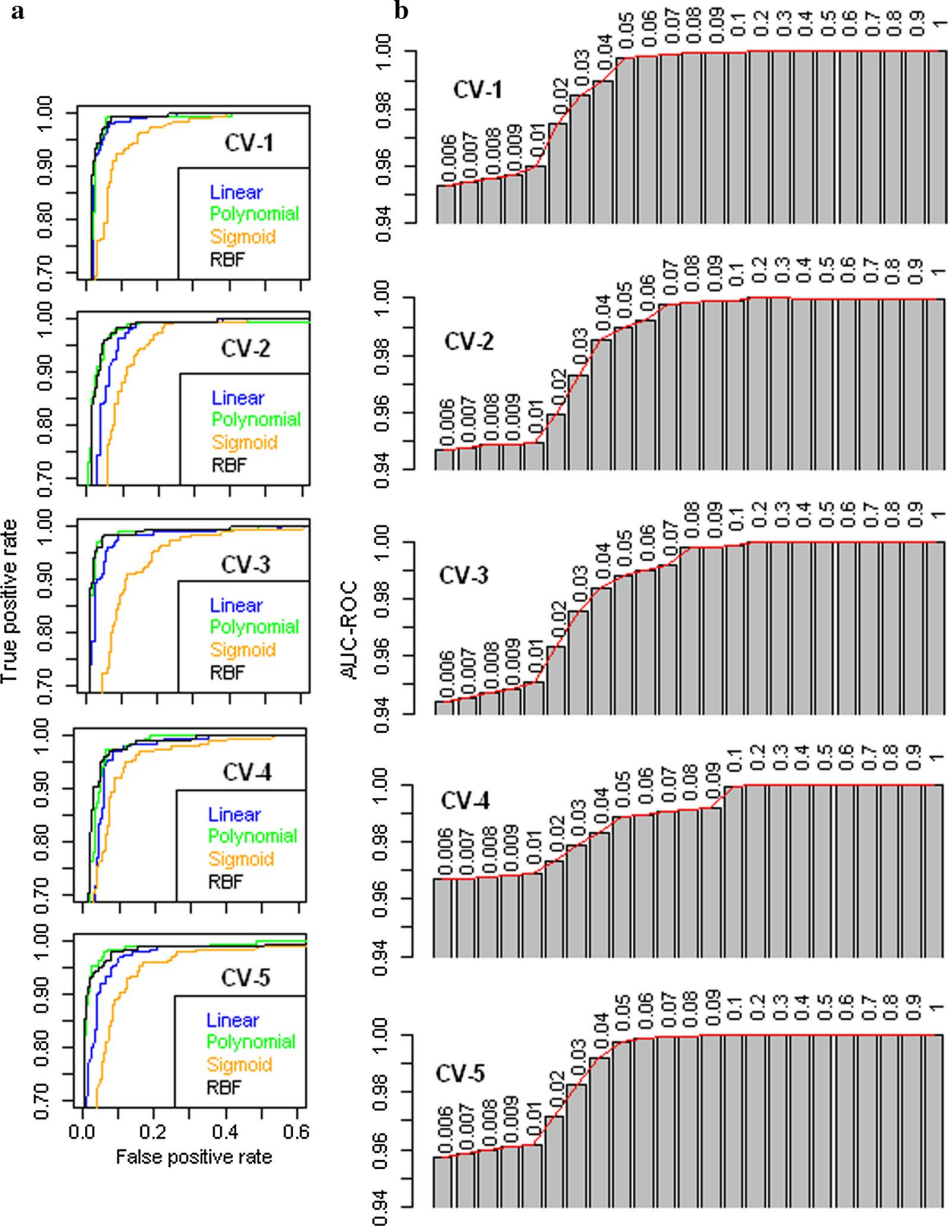
**a** ROC curves of SVM with linear, polynomial, sigmoid and RBF kernels in fivefold of the cross validation **b** *Bar plots* of AUC-ROC values for SVM with RBF kernel for different values of gamma (shown over each *bar*) in fivefold of the cross validation. SVM with polynomial and RBF kernels performed almost equally. Further, it can be seen that the AUC-ROC value of SVM with RBF kernel almost stabilized after 0.2 (value of gamma) in all the fivefold of the cross validation

ROC curves for four kernels across fivefold of cross validations are shown in Fig. 1a. It is observed that the performances of polynomial and RBF kernels are almost same and are superior to that of linear and sigmoid kernels in all the fivefold. Between polynomial and RBF, RBF kernel was selected because in most of the cases RBF required less number of hyperpameters and offered good generalization performance as compared to other kernels [4]. In case of RBF kernel, it is further seen that the values of AUC-ROC are increased with increase in the value of gamma from 0.006 to 0.2 and got stabilized thereafter (Fig. 1b) in all the fivefold. Thus, the value of gamma as 0.2 was considered as optimum and the final classification (training and testing) was performed using RBF kernel with this value of gamma in all the four species.

### Performance analysis of the proposed approach

**Fig. 2:**
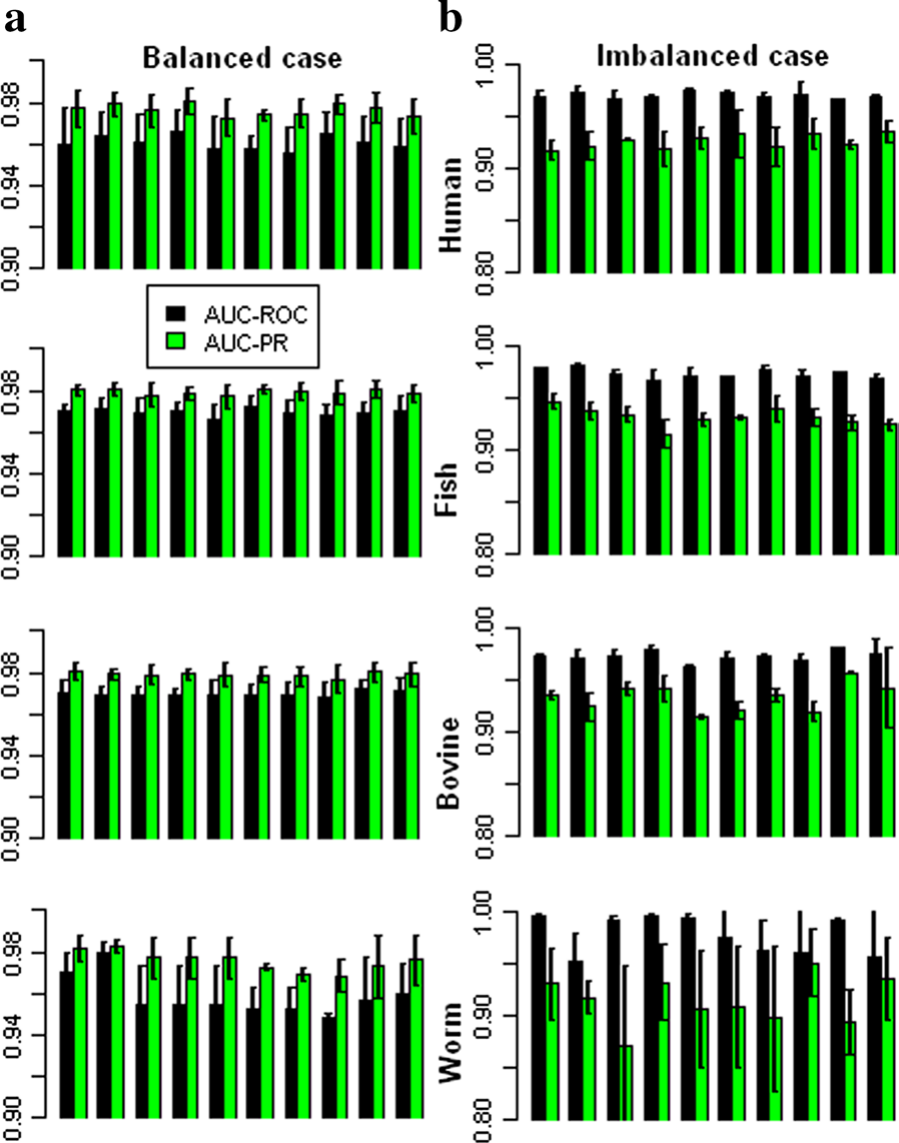
Estimates of AUC-ROC and AUC-PR for the proposed approach under balanced (**a**) and imbalanced (**b**) situations. Ten different *bars* represent ten different subsets, where each subset was drawn at random from the original data and AUC-ROC/AUC-PR was computed over fivefold of the cross validation

The AUC-ROC and AUC-PR computed over fivefold cross validation are shown in Fig. 2a (balanced case) and Fig. 2b (imbalanced case) for all the ten sets. It is observed that in case of balanced datasets the values of AUC-PR are respectively higher than that of AUC-ROC in all the four species. On the contrary, the values of AUC-PR are observed to be lower as compared to the respective AUC-ROC, while imbalanced datasets are used. In balanced case, it is seen that the values of AUC-PR are ~98 % and AUC-ROC are between 96 and 98 % in all the species, with exception in some sets of worm (Fig. 2a). In imbalanced situation, the values of AUC-ROC are found to be ~97 %, whereas the values of AUC-PR are found between 90 and 94 % (Fig. 2b). Besides, it seen that the values of AUC-ROC and AUC-PR are more consistent in case of human, fish and cattle as compared to worm. Furthermore, it is observed that the differences between the values of AUC-ROC and AUC-PR are higher in case of imbalanced situation as compared to the balanced case.

The values of AUC-ROC and AUC-PR averaged over fivefold and 10 sets are provided in Table 2. From Table 2 it can be seen that the values of AUC-ROC are >96 and >97 % for balanced and imbalanced datasets respectively in all the four species. It is further seen that the values of AUC-PR for the balanced dataset is >97 %, where as it is >93 % in case of imbalanced dataset, with exception in worm (<93 %). Besides, it is analyzed that the accuracies are consistent over the species.

**Table 2:** Performance accuracy of the proposed approach

Measure	Balanced				Imbalanced			
	Human	Cattle	Fish	Worm	Human	Cattle	Fish	Worm
AUC-ROC	96.05	96.94	96.95	96.24	97.21	97.45	97.41	98.06
AUC-PR	97.64	97.89	97.91	97.90	93.24	93.34	93.38	92.29

The performance of the proposed approach is measured in terms of AUC-ROC and AUC-PR in all the four species under both balanced and imbalanced situations. It can be seen that the values of AUC-ROC is almost similar in all the four species under both situations, whereas the values of AUC-PR are higher in balanced case as compared to the imbalanced situation

### Comparative analysis based on NN269 dataset

The performances of the existing and the proposed approaches are given in Table 3. From Table 3 it is observed that the value of AUC-PR is lowest in MM1-SVM (89.58 % AUC-PR). SVM with WD kernel achieved the highest AUC-ROC (98.50 %), whereas the AUC-PR is highest for the proposed approach (93.54 %). The AUC-ROC of the proposed approach (96.53 %) is observed to be 1.51, 1.97, 1.60 and 1.67 % lower than that of LIK-SVM, WD-SVM, WDS-SVM and EFFECT approaches respectively. On the other hand, the AUC-PR of the proposed approach is observed to be 0.89, 0.68, 1.07 and 0.73 % higher than that of LIK-SVM, WD-SVM, WDS-SVM and EFFECT approaches respectively. Since NN269 dataset is an imbalanced dataset, higher values of AUC-ROC of different approaches may not indicate their superiority over the proposed approach.

**Table 3:** Performance accuracies of different methods in predicting donor splice sites using NN269 dataset

Approaches	AUC-ROC	AUC-PR	Type of kernel used
MM1-SVM	97.62	89.58	Polynomial
LIK-SVM	98.04	92.65	Locally improved kernel
WD-SVM	*98.50*	92.86	Weighted degree kernel
WDS-SVM	98.13	92.47	Weighted degree shift kernel
EFFECT	98.20	92.81	–
Proposed	96.53	*93.54*	Radial basis function

It can be seen that WD-SVM achieved higher value of AUC-ROC as compared to the others, whereas the AUC-PR is highest for the proposed approach. MM1-SVM achieved lowest accuracies both in terms of AUC-ROC and AUC-PR

### Comparative analysis based on other datasets

Besides NN269 dataset, the performance of the proposed approach was also compared with that of other approaches using human, bovine, fish and worm datasets as mentioned in *collection and processing of splice site data.* The AUC-ROC and AUC-PR computed over fivefold cross validation are given in Table 4 (balanced case) and Table 5 (imbalanced case) respectively. In balanced dataset, the AUC-ROC of the proposed approach is seen ~1 % less than that of others (Table 4), whereas in imbalanced dataset the AUC-ROC of proposed approach is observed to be at par with that of other approaches (Table 5), in all the four species. Further, the AUC-PR of the proposed approach is observed to be approximately same with that of LIK-SVM, WD-SVM, WDS-SVM and EFFECT and ~1 % higher than that of MM1-SVM, for balanced dataset (Table 4). Whereas in imbalanced dataset, the AUC-PR of proposed approach is seen to be ~3 % higher than that of MM1-SVM and ~1 % higher than that of LIK-SVM, WD-SVM, WDS-SVM, EFFECT (Table 5), in all the four species.

**Table 4:** Estimates of AUC-ROC and AUC-PR of different methods for balanced dataset in predicting donor splice sites using human, bovine, fish and worm species

	Species	Approaches					
		MM1-SVM	LIK-SVM	WD-SVM	WDS-SVM	EFFECT	Proposed
AUC-ROC	Human	97.07	97.13	97.25	97.06	97.15	96.05
	Bovine	96.98	97.63	97.83	97.59	97.70	96.94
	Fish	97.24	97.34	97.68	97.53	97.59	96.95
	Worm	97.49	98.02	98.23	98.12	98.15	96.24
AUC-PR	Human	96.78	97.52	97.67	97.38	97.58	97.64
	Bovine	96.66	97.48	97.59	97.26	97.51	97.89
	Fish	96.85	97.42	97.67	97.39	97.49	97.91
	Worm	96.92	97.51	97.78	97.63	97.71	97.90

It can be seen that the values of AUC-ROC of the proposed approach are less as compared to that of others, whereas the values of AUC-PR for the proposed approach are at par with that of other approaches (except MM1-SVM), in all the four species

**Table 5:** Estimates of AUC-ROC and AUC-PR of different methods for imbalanced dataset in predicting donor splice sites using human, bovine, fish and worm species

	Species	Approaches					
		MM1-SVM	LIK-SVM	WD-SVM	WDS-SVM	EFFECT	Proposed
AUC-ROC	Human	97.32	97.61	97.73	97.30	97.42	97.21
	Bovine	97.57	97.89	97.93	97.65	97.70	97.45
	Fish	97.71	97.85	97.92	97.77	97.57	97.41
	Worm	97.99	98.26	98.51	98.30	98.45	98.06
AUC-PR	Human	89.95	92.23	92.36	92.17	92.41	93.24
	Bovine	90.02	92.13	92.39	92.16	92.42	93.34
	Fish	90.10	92.18	92.43	92.26	92.47	93.38
	Worm	89.10	90.27	90.89	91.53	91.67	92.29

It can be seen that the values of AUC-ROC of proposed approach are at par with that of others, whereas the values of AUC-PR for the proposed approach are little higher than that of other approaches, in all the four species

### Prediction server

**Fig. 3:**
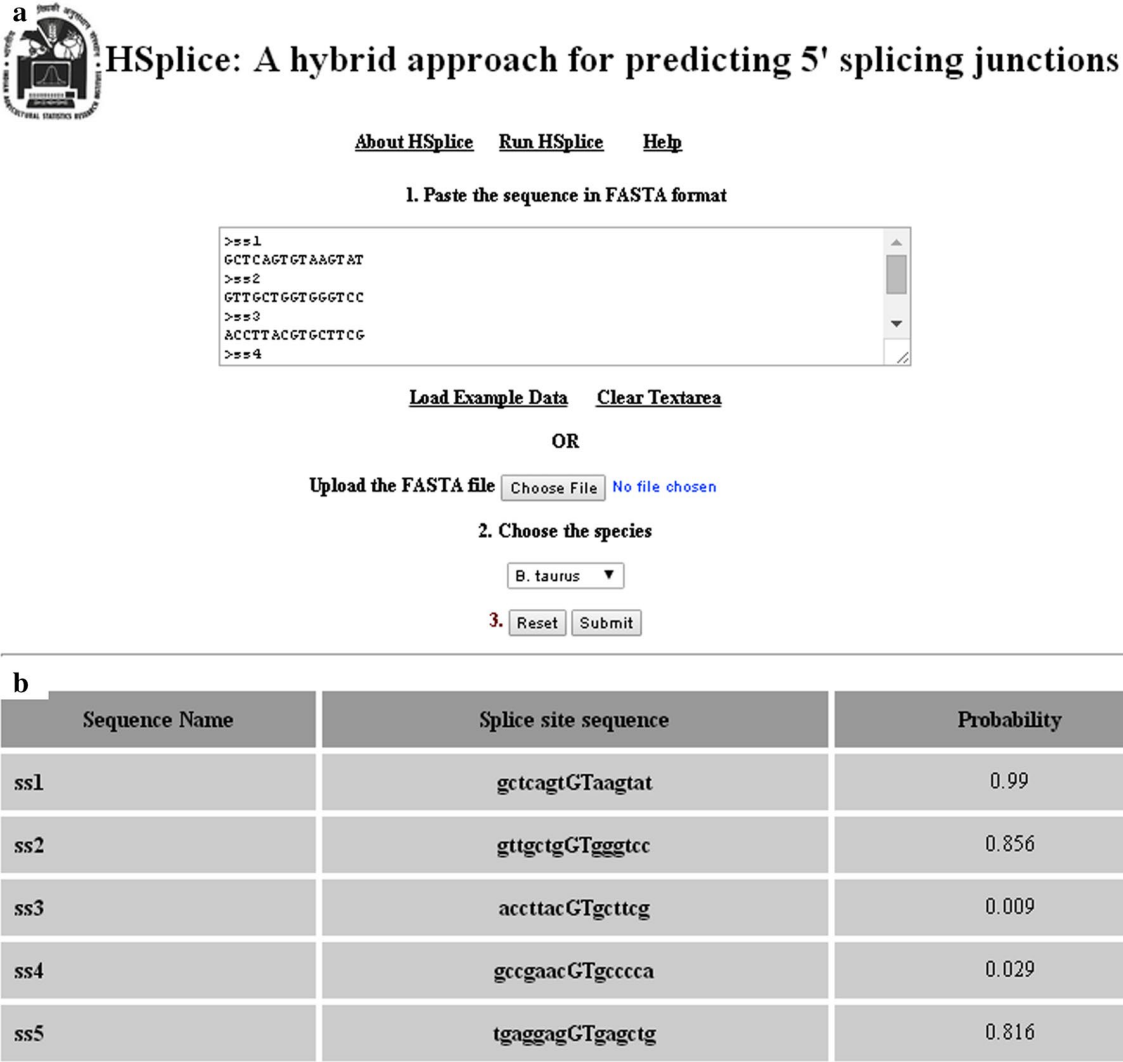
Snapshots of the server page (**a**) and result page after executing an example dataset (**b**) of the developed prediction server HSplice. The server has been trained with human, cattle and fish splice site datasets. The user has to supply only the test sequence for prediction of donor splice site for the species of his/her interest

Based on the proposed approach, we have developed an online prediction server “Hsplice” that can readily be used. This server has been trained with splice site datasets of *H. sapiens, B. taurus, D. rario,* and can be used for prediction of donor splice sites for these species. Due to lesser number of true splice sites (1000), the server is not trained for prediction of splice sites in *C. elegans*. The provision for both uploading the FASTA file as well as pasting the sequences in FASTA format is given. The server has been designed using HTML and PHP. Snapshots of the front page of the server and result page after executing a sample dataset are shown in Fig. 3a and b respectively. The results are displayed in three different columns i.e., 1st column: names of the respective FASTA sequences, 2nd: candidate splice site sequences of 15 nt and 3rd: probabilities with which the candidate splice site sequences are predicted as true (real) donor splice sites. Since the value of probability lies between 0 and 1, the value 0.5 can be considered as a threshold value and the candidate sequence predicted with probability >0.5 can be considered as real splice site. Higher is the probability more is the likelihood of a sequence to be predicted as true splice site. The HSplice is freely available at http://cabgrid.res.in:8080/hsplice.

## Discussion

In this investigation, we proposed an approach for the prediction of donor splice sites in four different species by using 15 nt long sequence motifs. The proposed approach was executed in two phases. In the first phase, a new set of features were generated and informative features were screened by using F-score. In the second phase, the informative features were used as input in SVM classifier for predicting true and false donor splice sites.

Three different types of features i.e., positional, dependency and compositional features were used in this study. The positional features were similar to the scores of WMM and Shapiro-Senapathy score, whereas the dependency features were similar to the scores of earlier developed probabilistic approaches i.e., WAM and SAE. WMM and Shapiro-Senapathy scores did not take into account the positional dependencies. In WAM, the dependencies among the adjacent positions are taken into account, whereas in SAE all possible di-nucleotide dependencies are considered [6]. In compositional features, the compositions of di-, tri- and tetra- nucleotides were considered because they have been found useful in discriminating the true splice sites from false ones [3, 4, 30]. Among the three types of features, the F-score was found to be higher in positional and dependency features as compared to the compositional features (Table not reported here), and this is probably due to the partial conserved-ness of nucleotides surrounding the splicing junctions. In probabilistic approaches viz., WMM, WAM, MEM, MDD, the true splice sites have only been used for computing the scores of candidate splice site sequences. Also, in MM1-SVM, only the true sites are used for generating the feature vectors. However, both true and false splice sites sequences need to be trained for the prediction of splice sites [8]. Therefore, positional and dependency features were computed in two different ways i.e., by using only true sites ($f_{1}^{P \times T} ,f_{3}^{P \times T} ,f_{5}^{D \times T} ,f_{7}^{D \times T}$) and by using both true and false splice sites ($f_{2}^{P \times TF} ,f_{4}^{P \times TF} ,f_{6}^{D \times TF} ,f_{8}^{D \times TF}$). It was found that the F-scores of the features computed by taking both true and false splice sites were higher as compared to that of features extracted solely based on the true splice sites. The motivation behind using these probabilistic scores as the features are (1) difficulty in determining the threshold values in case of probabilistic approaches [1], and (2) easy in determining the threshold values in machine learning based classifiers (i.e., 0.5, in terms of probability).

Out of 344 features, only 49 features were selected through F-score and subsequently used as input in the classifier. The classification task was performed through SVM by using RBF as kernel. Both balanced and imbalanced datasets were used to assess the performance of the proposed approach, which was measured in terms of AUC-ROC and AUC-PR averaged over five-fold cross validation. In balanced case, the AUC-ROC and AUC-PR were found to be ~96 and 97 % respectively, whereas in imbalanced situation these values were ~97 and ~93 % respectively (Table 2). Further, it was analyzed that the values of AUC-ROC were similar both in balanced and imbalanced situation. Besides the difference between the values AUC-ROC and AUC-PR was higher in imbalanced datasets. This may be due to that the AUC-ROC is independent of class ratio, whereas the AUC-PR is influenced by the presence of class-imbalance in the datasets [3]. The values of AUC-ROC and AUC-PR were also observed to be consistent over the fivefold of cross validation with some exceptions in case of worm dataset.

The proposed approach was further compared with the state of art splice site prediction methods i.e., MM1-SVM, LIK-SVM, WD-SVM, WDS-SVM and EFFECT. The comparison was made by using an independent dataset i.e., NN269 dataset. In terms of AUC-ROC, WD-SVM achieved higher accuracy as compared to the others and the accuracy was ~2 % higher than that of proposed approach. On the other hand, proposed approach achieved 93.54 % AUC-PR, which was ~1 % higher than that of WD-SVM. Further, WD-SVM achieved higher AUC-PR as compared to other approaches barring proposed approach. Besides proposed approach, all others achieved AUC-ROC ~98 % and AUC-PR of ~93 % (except MM1-SVM). Since, AUC-PR is thought to be a better measure than AUC-ROC in case of imbalanced dataset, it can be said that the proposed approach can be used as a complementary method to the other methods for the prediction of donor splice sites.

Besides NN269, the AUC-ROC of the proposed approach was also found to be ~1 % less than that of MM1-SVM, LIK-SVM, WD-SVM, WDS-SVM and EFFECT, while comparison was made using balanced datasets of human, bovine, fish and worm. On the other hand, the AUC-PR of the proposed approach was found to be ~3 % higher than that of MM1-SVM and ~1 % higher than that of LIK-SVM, WD-SVM, WDS-SVM and EFFECT, while imbalanced datasets of human, bovine, fish and worm were used. Thus, the proposed approach is believed to supplement the existing splice site prediction approaches.

The number of features used in the proposed approach is invariant to the length of the sequence, whereas in the existing approaches like MM1-SVM, Bayes-SVM [11], FDTF [8], DS-SVM [1] the number of features increases with the increase in the length of sequence. The proposed approach showed consistent performance by using a shorter window size of 15 nt long, and therefore it may be suitable for detecting splice variants in short reads generated from sequencing technologies. Since the accuracies were found to be consistent over human, cattle and fish similar accuracies can be expected in other vertebrates. The developed web server HSplice (http://cabgrid.res.in:8080/HSplice) can be used by the researcher community for prediction of donor splice sites easily.

## Conclusions

This paper presents a computational approach for the prediction of donor splice sites using SVM with a different set of features that have not been used in earlier studies. The proposed approach was tested on human, cattle, fish, worm datasets and found to achieve an acceptable level of accuracy in all the species. The proposed approach was also found to be comparable with the existing state-of-art prediction methods, and thus can complement to the existing methods. The HSplice will help enable the user for easy prediction of donor splice sites.

## Abbreviations

SVM: support vector machine
HS3D: homo sapiens splice sites dataset
MEM: maximum entropy modeling
MDD: maximal dependency decomposition
WMM: weighted matrix model
WAM: weighted array model
MM1: Markov model of 1st order
TSS: true splice sites
FSS: false splice sites
ROC: receiving operating characteristics
PR: precision-recall
AUC-ROC: area under ROC curve
AUC-PR: area under PR curve
LIK-SVM: SVM with locally improved kernel
WD-SVM: SVM with weighted degree kernel
WDS-SVM: SVM with weighted degree shift kernel
RBF: radial basis function
